# Ganglion Cell Complex Analysis: Correlations with Retinal Nerve Fiber Layer on Optical Coherence Tomography

**DOI:** 10.3390/diagnostics13020266

**Published:** 2023-01-11

**Authors:** Aurelian Mihai Ghita, Daniela Adriana Iliescu, Ana Cristina Ghita, Larisa Adriana Ilie, Alexandru Otobic

**Affiliations:** 1 Ophthalmology Department, Bucharest University Emergency Hospital, 169 Splaiul Independenței Street, 050098 Bucharest, Romania; 2 Department of Physiology, “Carol Davila” University of Medicine and Pharmacy, 8 Eroii Sanitari Bld., 050474 Bucharest, Romania; 3 Ocularcare Ophthalmology Clinic, 128 Ion Mihalache Bld., 012244 Bucharest, Romania

**Keywords:** ganglion cell complex, retinal nerve fiber layer, optical coherence tomography, glaucoma stages

## Abstract

The aim of this review is to analyze the correlations between the changes in the ganglion cell complex (GCC) and the retinal nerve fiber layer (RNFL) on optical coherence tomography in different possible situations, especially in eyes with glaucoma. For glaucoma evaluation, several studies have suggested that in the early stages, GCC analysis, especially the thickness of the infero and that of the inferotemporal GCC layers, is a more sensitive examination than circumpapillary RNFL (pRNFL). In the moderate stages of glaucoma, inferior pRNFL thinning is better correlated with the disease than in advanced cases. Another strategy for glaucoma detection is to find any asymmetry of the ganglion cell–inner plexiform layers (GCIPL) between the two macular hemifields, because this finding is a valuable indicator for preperimetric glaucoma, better than the RNFL thickness or the absolute thickness parameters of GCIPL. In preperimetric and suspected glaucoma, GCC and pRNFL have better specificity and are superior to the visual field. In advanced stages, pRNFL and later, GCC reach the floor effect. Therefore, in this stage, it is more useful to evaluate the visual field for monitoring the progression of glaucoma. In conclusion, GCC and pRNFL are parameters that can be used for glaucoma diagnosis and monitoring of the progression of the disease, with each having a higher accuracy depending on the stage of the disease.

## 1. Introduction

The aim of this review is to provide an insight into the interpretation of the ganglion cell complex (GCC) using the optical coherence tomography (OCT). The analysis of GCC parameters according to different possible situations, with a focus on glaucomatous eyes, is reported. The OCT is a noninvasive imaging method that has been widely used for the evaluation of structural abnormalities that are affecting the optic nerve head, retinal nerve fiber layer (RNFL) of the peripapillary region and the macular area including the GCC [1,2,3]. The circumpapillary RNFL (pRNFL) has long been used as an OCT parameter for the evaluation of glaucoma and other ocular diseases affecting the optic nerve. Nevertheless, by analyzing only the axons of the retinal ganglion cells, impairments on the ganglion cell layers (GCL) and the inner plexiform layers (IPL) are missed [4,5,6]. In addition, in the central retina, the thickness of the GCL is significantly larger than that of the nerve fiber layer, with the ganglion cell body rows having a 10-fold increased thickness compared to the axons layer [7]. The evaluation of the macular GCC is now available by using automatized segmentation of the retinal layers with the introduction of newer OCT technology [8]. While the time-domain OCT devices were able to analyze only the total macular thickness, which is not correlated with a good accuracy in diagnosing glaucoma patients, the recent spectral-domain OCT (SD-OCT) devices allow the measurement of all three layers that form the macular GCC: macular retinal nerve fiber layer (mRNFL), GCL and IPL [9]. GCC is one of the parameters used for the early-stage diagnosis of the glaucomatous disease, the evaluation of the disease progression and monitoring the advanced stages [4,10,11]. Given the fact that the first segment affected regarding the ganglion cells is the IPL (synapses at this level are initially damaged), followed by the GCL, GCC may prove to be a better diagnostic tool for the early glaucomatous lesions. The process of mitochondrial splitting is one of the initial changes observed that demonstrates the damage on the dendritic layer of the ganglion cells (IPL) [12]. In addition, it has been found that GCC is a superior parameter in cases of ocular hypertension that were associated with structural changes like tilted disc or atrophy surrounding the optic nerve, in which the pRNFL could not be properly determined on the OCT [13]. On the other hand, the changes of GCC are nonspecific, like the pRNFL, and can be altered in other ocular diseases such as multiple sclerosis, ocular ischemia, diabetes mellitus and toxic syndrome [14,15,16].

## 2. Acquisition Technique of GCC

The macular GCC quantifies the thickness between the internal limiting membrane and the outer border of IPL. GCC analysis evaluates the inner layers of the retina and includes GCL, IPL and facultative mRNFL (depending on the OCT machine). There are different acquisition settings available for the evaluation of GCC using the SD-OCT. They have distinct methods for the analysis of the GCC layers. Even though there are more than 10 SD-OCT devices available, a general presentation of the OCT machines that can analyze GCC includes Cirrus HD-OCT (Carl-Zeiss Meditec, Dublin, CA, USA), Spectralis (Heidelberg Engineering, Heidelberg, Germany), RTVue-100 (Optovue Inc., Fremont, CA, USA), RS-3000 SD OCT (Nidek, San Jose, CA, USA) and 3D OCT (Topcon, Livermore, CA, USA).

The characteristics of the GCC examination are speed of acquisition, examined area (dimension and centered or not on the fovea) and number of layers considered in the examination [4]. All these perform GCC scans from the center of the macula to an area that varies between a 6 × 6 mm^2^ grid (Cirrus HD-OCT, Carl Zeiss) to a 9 × 9 mm^2^ grid (RS-3000 SD-OCT, Nidek). Approximately half of the total number of retinal ganglion cells are centered in 5 mm of the foveal zone. The topographic distribution analysis of the ganglion cells has shown a much higher density in the central macular area compared to the more peripheral retina [17]. Depending on the manufacturer, the acquisition rate can vary between 26,000 A-scans per second (for the RTVue-100, Optovue) to 85,000 scans per second for the Spectralis OCT2 module (Heidelberg Engeneering) [18]. Such a high scan rate provides data for the 3D imaging of the interest area. The progression analysis software is another useful tool that enables the comparison of the data available at the baseline and the follow-up value to observe a progressive decrease of the GCC thickness. The mean values of the GCC thickness are around 100 µm, conditioned by the OCT type. Although the available OCTs scan the macular region for the measurement of GCC, the segmentation protocol is different between manufacturers.

The Cirrus HD-OCT (Carl-Zeiss Meditec) scans at a rate of 27,000 A scans/sec and analyzes both the IPL and the GCL. The algorithm of Cirrus OCT segments and identifies the outer boundary of the IPL and the external limit of the nerve fiber layer. The data are collected from an area of 14.13 mm^2^ with the center at the level of the fovea. The results are presented as 6 sectorial thickness maps that show the absolute values and probability colored maps, resulted through comparison with a normative database. The green, yellow, or red of the probability maps show the normal (*p* = 5–95%), borderline (*p* = 1–5%) and, respectively, abnormal values (*p* ˂ 1%) [4,19,20].

Spectralis (Heidelberg Engineering) OCT is able to segment and quantify each of the three layers composing the GCC. The data are acquired from a perifoveolar volume scan (30 × 25°), consisting of 61 B scans. The retinal thickness maps are analyzed from 9 macular areas centered on the fovea. The areas are divided into rings that contain the superior, inferior, nasal and temporal zones. The retinal maps with colorimetric scale are displayed. The posterior pole asymmetry protocol of Spectralis OCT can present asymmetric patterns between the macular regions of the eyes [21].

The RTVue-100 (Optovue Inc.) system maps an area of 7 × 7 mm^2^, centered 0.75 mm temporal to the fovea. The software measures the GCC thickness formed from the combination of mRNFL, GCL and IPL [9]. The acquisition rate is of 26,000 A scans/sec. The results are presented as a colorimetric scale map of the GCC thicknesses, a deviations map that shows the percentage of the GCC loss compared to the OCT’s normative database and a significance map. The GCC scanning protocol utilizes a 7 mm horizontal scan line, together with 15 vertical 7 mm lines. Two parameters also result from the acquired data: the focal loss volume (FLV) and the global loss volume (GLV), which appear to be more accurate in the diagnosis than the average GCC loss [4,22].

The RS-3000 SD OCT (Nidek) has a depth resolution of 7 µm and of 20 µm in the transverse section of the tissue. The GCC is scanned from an area of 9 × 9 mm^2^ centered on the fovea. From this, the central 1.5 mm diameter circle is excluded from the analysis. The scanning density consists of a horizontal line scan of 512 A-scans and a vertical line of 128 B-scans. The collected data are displayed as superior and inferior thickness values [23].

The 3D OCT from Topcon has a scanning rate of 50,000 scans/sec and consists of 512 horizontal A scan lines and 128 vertical A scan lines. It has a depth resolution of 6 µm and a lateral resolution of 20 µm. The scanned macular area is 6 × 6 mm^2^ and provides fundus imaging. The software can analyze the mRNFL, GCL together with the IPL and GCC. The results are compared to the normative data and presented as color-coded maps [4,24].

## 3. OCT in Neurological and Vessel Diseases

GCC and RNFL have been extensively studied in recent decades. Many neurological and ophthalmological diseases involve the optic nerve and affect GCC and pRNFL. For this reason, GCC changes are not specific only for glaucoma. All the modern OCT machines measure the pRNFL and the GCC quantitatively and compare the results with the normative data. Advanced analysis software will even compare the superior and inferior parts of the median raphe to detect the early changes in patients with optic nerve diseases.

GCC is similar to pRNFL regarding glaucoma detection and can be a useful tool in monitoring progression. Donaldson et al. compiled an exhaustive study of neuro-ophthalmology diseases and their OCT results [25]. As long as there is no coexisting macular damage or edema, the GCL measurements can be used as a tool to monitor the optic nerve insult in cases of papillary edema, because the increase of pRNFL will mask any axonal loss. In addition, GCC is useful in cases in which the pRNFL measurements are unreliable (high myopia, small or tilted optic nerve head) [25].

In some cases of branch retinal artery occlusion, the altitudinal visual field (VF) defects can be similar to those found in non-arteritic anterior ischemic optic neuropathy (NAION). The inner nuclear layer (retinal ganglion cell body) atrophy is always expected after a retinal arterial occlusion, but not after NAION, thus easing the diagnosis [26].

At onset, optic neuritis is associated with a mild pRNFL thickness increase, while the GCL and IPL remain normal. An episode of optic neuritis is followed by thinning of the pRNFL and ganglion cell—inner plexiform layer (GCIPL), which is more pronounced than pRNFL and starts approximately 2 weeks after the onset of symptoms. Even though the retrobulbar portion of the optic nerve is the main location of the optic neuritis damage, retrograde axonal degeneration appears quickly, being the highest in the first month and leading to the GCIPL thinning. The normal GCIPL with no asymmetry between eyes at 4–6 weeks after the onset of the symptoms usually makes the optic neuritis diagnosis improbable [25].

Studies have shown that there is a correspondence between the neurologic disease location and the pRNFL and GCC loss. The lesions that are closer to the globe and before the lateral geniculate nucleus tend to develop a fast and severe pRNFL and GCC loss, whereas the diseases that involve the optic radiations and the occipital cortex will cause a mild, unspecific RGC loss that is not proportional to the injury [25].

The optic chiasm lesions will usually result in the damage of the decussating nasal fibers, followed by the retrograde degeneration of the ganglion cell axons and the binasal thinning of the GCIPL in about 4 weeks. It has been noted that in cases of suspicion of chiasmal lesions, 50% of patients will have normal VF, but GCIPL thinning. This suggests that the decrease of GCC is prior to VF changes and may be a good indicator of the need for surgical treatment [27]. In addition, the loss of normal nasal–temporal asymmetry (the nasal GCIPL is thicker than the temporal GCIPL) can be an early structural change in parasellar lesions. The absence of GCIPL thinning in patients with chiasmal lesions is a good prognostic factor for the postoperative improvement of VF [28].

Similarly, optic tract lesions will cause homonymous GCIPL thinning respecting the vertical midline [29].

The lesions involving the optic radiations and the occipital lobes will be followed by trans-synaptic degeneration with varying rates of RNFL and GCIPL thinning, which seems to be the highest in the first 2 years after the insult [30].

## 4. Variation of GCC and RNFL with the Stages of Glaucoma

A significant difference in the structure-function correlation can be found in different stages of glaucoma (Table 1), and therefore the progression of glaucoma detected on OCT, similar to the diagnosis of glaucoma, must be evaluated in the context of clinical examination and evaluation of the VF. Clinical trials indicate that structural abnormality may precede functional abnormality in some cases, while the reverse is true in others. Although OCT plays an important role in glaucoma evaluation, staging is performed using the MD of the VF according to the guidelines. Conventional pRNFL is an indirect method for the assessment of retinal ganglion cells (RGC), which is a surrogate indicator for the affected cells in glaucoma. Some studies have shown that pRNFL is statistically non-inferior in the detection of glaucoma, and paradoxically superior to GCC analysis. This fact implies two points regarding GCC and pRNFL. One aspect is related to the GCC analysis that evaluates the central or macular RGCs while peripheral RGCs are neglected. For many patients with glaucoma, the loss of RGC starts in the periphery of the macula and even outside of the examination area. This fact, combined with the limitations of the GCC analysis, led to a decreased cell count loss in some patients. Experimental studies have observed that the loss of GCC is uneven at the level of the retina [4]. Glaucoma is a chronic disease and, for many patients, the loss of RGCs is relatively slow but steady, which allows the loss of RGCs to be quite similar to the loss of pRNFL. Finally, the pRNFL evaluates the axons from all the RGCs, which allows the identification of any loss of RGCs. However, the evaluation is quite rough because the parameters evaluate sectors of pRNFL and not individual cells.

### 4.1. Early Stage

In early stages of glaucoma, several studies tried to find out which OCT or ONH (optic nerve head) parameter is the best diagnostic tool [31,32,33,34]. According to the study performed by Lisboa et al., pRNFL (average, inferior hemisphere average and inferior quadrant average) is considered of higher diagnostic power compared to macular scans (GCC or total thickness) and to ONH parameters [11]. In the case of patients with VF mean deviation with a better index than −6 dB, studies have reported contradictory results regarding which SD-OCT parameter is superior. Moreno et al. concluded that statistically, the inner retina layer thickness (GCC) is equal or better than the pRNFL thickness analysis [35]. However, at the same time, since many cases are not identified by both protocols, this study admits that GCC and pRNFL scans should be used in a complementary manner.

### 4.2. Moderate Stage

Due to the asymptomatic nature of early glaucoma often leading to delayed diagnosis, the usual first presentation is in a moderate to advanced stage of the disease. Which OCT parameter is more suitable for follow-up is of great importance, especially in the case of a chronic, potentially blinding disease, for which treatment costs increase with severity [36]. Studies have shown that there is a correlation between the extent of the MD value on VF and the GCIPL or RNFL utility in the progression assessment. It was statistically demonstrated that in early and moderate glaucoma, inferior RNFL thinning is better correlated with the disease than in advanced cases (floor effect). In advanced cases, superior GCIPL thickness tends to be preserved more than in early and moderate cases, correlating with the profile of the central VF island [37].

### 4.3. Advanced Stage

In advanced stages, the thickness of RNFL and GCC is not zero because glaucoma affects the RGC but not the support cells (glial cells). Usually, the thickness of GCC in late stage glaucoma is around 55 to 70.7 μm [38,39,40,41] and of pRNFL between 44.9 and 53.7 μm [41,42,43,44]. We noted this residual thickness in late stage glaucoma as the floor effect that structurally represents the glial tissue and blood vessels. Paradoxically, in advanced glaucoma, the floor effect is achieved earlier by the RNFL and later by GCC [45]. To study the progression in advanced glaucoma it is useful to examine the superior temporal area of GCC, which is the last segment completely affected. An explanation for this paradox is related to the attempt of the residual RGC to build new synapses to compensate the loss of the large number of RGC. These new synapses lead to a decrease of the GCC thickness slope. The floor effect is firstly achieved by pRNFL, then by the inferior segment of GCC and finally by the superior segment of GCC [37]. Therefore, the papillomacular bundle is relatively resistant to changes in the glaucoma disease until the advanced stages, and higher rates of macular thinning are specific to the progressive disease [46]. Moreover, recent studies conducted by Shin et al. and by Zhang et al. confirmed that the GCIPL thinning is a good predictor of the disease progression regardless of the glaucoma severity, contrary to pRNFL, which failed to detect progression in moderate to advanced glaucoma [47,48]. Correct assessment has solid clinical implications because the treatment decisions are based on the progression status of the patient. This decision is even more important in the case of advanced glaucoma due to the considerable risk of functionally impairment or blindness and the costs borne by these conditions [36].

## 5. Follow-Up of Glaucoma by Considering the GCC Thickness Change

A study conducted by Lee et al. [49] compared the GCIPL thinning in normal subjects, primary open angle glaucoma (POAG) patients and pseudoexfoliation (PEX) glaucoma patients treated with topical medication over a period of more than 3 years. The results showed a significantly faster GCIPL thinning in the PEX group when compared with the other two groups (−0.31 µm/y normal eyes, −0.49 µm/y POAG, −1.46 µm/y PEX glaucoma). Regarding the POAG group, using an arbitrary cutoff value of 21 mmHg of the intraocular pressure (IOP) when classifying the participants, no significant difference between the groups was observed. One explanation for this situation is the fact that normal tension glaucoma (NTG) and hypertension glaucoma (HTG) are part of a continuous spectrum of the same disease, and the arbitrary IOP cutoff value could not differentiate enough between the two conditions with respect to the GCIPL thinning. By comparing the POAG patients with normal subjects, this study found a higher rate of the GCIPL thinning in glaucoma patients, although the results were not statistically significant. Some reasons for this outcome are the grouping of all the POAG stages and the relative short duration of the study. Other GCIPL parameters, such as minimum, superonasal and inferonasal thickness have a faster thinning with a stronger statistical significance compared to the average GCIPL thickness [49]. GCIPL seems to be an effective tool for progression detection. Most of the GCIPL parameters displayed grades of thinning. Nevertheless, the best results for discriminating progression were provided by the global and minimum thickness GCIPL parameters for the affected hemifield and by the temporal sectors thinning rates [50]. Studies that investigated the normal rate of GCIPL age-related thinning stated it to be −0.318 µm/y, a rate that could be used in glaucoma progression assessment [51]. Other studies found an age-related GCIPL thinning of −0.53 ± 0.36 µm/y for the healthy subjects, in line with the previous studies [52].

## 6. Other Criteria Based on GCC for Glaucoma Diagnosis

### 6.1. Macular Vulnerability Zone

Hood et al. conducted a study which concluded that there is an asymmetry between the inferior and superior fields on the OCT macular scans, and that the greater GCC damage is located inferiorly in glaucoma patients [53]. Furthermore, this study underlined that the GCC thinning was more pronounced on the temporal side of the fovea. This study, as well as other newer studies, describe the existence of a sensitive pRNFL region of the disc, stretching from 7 o’clock to 8 o’clock, which is prone to glaucoma damage. Clinically, disc hemorrhages in this region of pRNFL have been described and functionally as an arcuate defect found in the upper macular VF. Loss of pRNFL is associated with a decrease in the GCC thickness in the inferior temporal region of the macula named the “macular vulnerability zone” (MVZ). The strategy for glaucoma detection is to find the asymmetry between the superior and inferior sectors of the macula. This starts with the anatomical asymmetry of the retinal fibers’ distribution, demonstrated by several studies which show that the temporal part of the horizontal raphe does not strictly follow the horizontal meridian, having a slope, on average, 10 degrees above the horizontal meridian [54,55,56]. The reason for this initial inferior loss of GCC and then of the inferior RNFL compared to the superior area, is related to the higher fragility of the inferior lamina cribrosa and to the higher density of fibers in the inferior segment compared to the superior one [57,58,59,60]. The fragility of the inferior lamina is due to the faint connective tissue and to the large pores that could be secondary to the high density of fibers in the inferotemporal sector of the papilla [58,59,60,61]. The high-density fibers are related to the lower position of the fovea compared to the papilla. This results in the asymmetrical distribution of the inferior arcuate bundle when compared to the superior arcuate bundle [53,62]. Because of this asymmetry, the axons of RGC of the superior part of the macula correspond to the temporal segment of the papilla, while the axons of RGC of the inferior area of the macula correspond both to the temporal and to the inferior segment of the papilla. The fact that the inferotemporal segment of the papilla, known as the MVZ, is particularly susceptible to glaucoma damage and corresponds to the inferotemporal part of the macula, as seen above, explains why the first loss of GCC is in the inferotemporal area [53,62]. These studies also underline that the superior and cecocentral macular regions are better protected from glaucomatous damage when compared to the inferior part of the macula. The cecocentral RGC projects axons to the temporal quadrant of the disc where the ultrastructural characteristics of the lamina cribrosa could be protective against early glaucoma damage [63,64].

Other studies that focus on MVZ conclude that the most susceptible regions for the glaucomatous thinning of GCIPL are the inferior, inferotemporal and superotemporal sectors of the macular segmentation in Cirrus-OCT, and offer the best discriminatory performance [65].

### 6.2. Inferior-Superior Asymmetry

As a consequence of the MVZ, several studies focused on the superior–inferior asymmetry for an accurate detection of glaucoma, especially in the early stage. Comparing the asymmetry between the inner retinal layers of the upper and lower segments of the macula in preperimetric glaucoma patients and controls, the study of Takemoto et al. concluded that the asymmetry of GCIPL between the macular hemifields has a better ability to diagnose preperimetric glaucoma than the RNFL thickness or the absolute thickness parameters of GCIPL [66]. This study is consistent with the results of Yamada and Kim [67,68]. In addition, studies identified a diagnostic difference value of about 8 μm of GCIPL between the upper and lower segments in preperimetric glaucoma patients, and hypothesized that the OCT thickness asymmetry of the inner retinal layers is an initial sign of glaucoma onset [66]. Other studies suggested 20 μm as the diagnostic cut-off value, but in these cases, the disease was advanced and associated with perimetric defects [69].

### 6.3. Interocular GCC Asymmetry

It is usually considered that healthy organ pairs display symmetric architectural features. With reference to GCC, a highly symmetric distribution of RGC axons and cell bodies has been confirmed when comparing interocular and interhemispheric parameters of healthy eyes [69,70]. Observing that the clinical progression of glaucoma is often interocular and interhemispheric asymmetric, a significant asymmetry of retinal parameters, when compared to healthy eyes, may suggest that the glaucoma disease has emerged. Studies uncovered significant statistical results (*p* < 0.001) for the interocular difference of the average GCIPL thickness, and of the minimum sectoral GCIPL thickness in the diagnosis of glaucoma. The area under the receiver operating characteristic curve (AUROC) of the average GCIPL interocular thickness difference was 0.8 [71,72,73,74]. A guideline of the normal GCIPL interocular difference tolerance limit was considered to be between −4.10 µm and ±5.00 µm. On the other hand, Lee SY also suggested that significant GCIPL interocular asymmetry can be caused by differences in the axial length of the eyeball [75]. In addition to the previous studies, a second study lead by Lee SY et al. compared the interocular inner macular thickness using swept source OCT, and observed that the average negative interocular GCIPL thickness difference was significantly higher in the glaucoma group than in the normal subjects’ group [69]. Other studies have confirmed that the interocular asymmetry of GCC is associated with the asymmetry of other parameters commonly used in glaucoma as ONH or pRNFL [69,71,76,77,78,79,80,81]. The high asymmetry of GCC and of the related parameters is associated with high glaucoma progression.

One exception from the interocular asymmetry in glaucoma is PEX. A study by Aydin et al. that compared to the unilateral PEX syndrome eyes, the fellow eyes of the patients and the healthy eyes underlined that almost all the GCC parameters, except for the inferior and inferonasal quadrants, were found to be significantly thinner in the PEX syndrome and fellow eyes compared to the healthy eyes [82]. Regarding the interocular GCC asymmetry, no significant difference was found, confirming that clinically, the unilateral PEX syndrome is truly a bilateral disease, a result strengthened by ultrastructural studies on unilateral PEX syndrome [83]. Careful follow-up for early glaucoma damage detection should be emphasized.

Newer studies tried to test if the derivative indices of the inter-eye asymmetry, like the GCC global loss volume percentage (GLV%), the GCC focal loss volume percentage (FLV%) or the perfused vessel density (PVD), are of any diagnostic use in cases of early-moderate POAG [84]. They determined that the highest diagnostic sensitivity for the inter-eye asymmetry indices is for FLV%, followed by GLV% and by more common indices like average GCC, inferior hemifield GCC, superior hemifield GCC and average RNFL. PVD asymmetry proved to be of modest POAG diagnostic use, with AUROCs significantly lower than the retinal structural parameters [85]. The reason why FLV% and GLV% perform well in early–moderate POAG lies in the fact that they calculate the GCC volume loss for varying levels of focality, in such a way that even when the average GCC thickness is within normal limits, early focal loss can be a sign of glaucoma disease [86]. This study marks the need to explore other GCC parameters in the search for the early diagnosis of glaucoma (Table 2).

## 7. The Comparison of GCC with Other Ophthalmologic Parameters in the Detection and Evaluation of Glaucoma Patients

There are plenty of methods to evaluate glaucoma: visual field, funduscopic examination, stereoscopic photography of the optic disc, optic nerve fiber analysis and others. Some investigations are considered standard for the evaluation of glaucoma patients, while others are new and not yet used regularly in diagnosis. The GCC analysis is considered a newer investigation method used by doctors in order to detect glaucomatous damage. Theoretically, the GCC is more sensitive than other parameters because it directly assesses the RGCs affected by glaucoma. However, for a better confidence in the GCC analysis, a series of studies have been available in the last decade, which compare the GCC with the other types of parameters [86]. When comparing GCC with RNFL, several aspects can be observed, such as the following: in different studies, no homogeneity can be noted in the glaucoma stages assessment by different OCT devices used for evaluating GCC. In addition, the percentage of RGC evaluated by the OCT equipment from the total RGC number is different, and the formula used to associate the changes of RGC with pRNFL is not the same.

### 7.1. The Comparison of GCC with RNFL

#### 7.1.1. The Value of GCC Compared to RNFL in Different Stages of Glaucoma

GCC and pRNFL are similar for detecting glaucoma, without statistical differences for the moderate and advanced stages. For the early glaucoma, the GCC analysis was suggested to be more sensitive than pRNFL for glaucoma detection [87,88,89]. Not all of the studies agree on the difference between GCC and pRNFL in detecting glaucoma, and they even assert the opposite [38,39]. For diagnosis in the early stages, the thickness of the infero and especially that of the inferotemporal GCC layers must be inspected. These changes in the GCC examination seem to be a better parameter than pRNFL for detecting early glaucoma and preperimetric glaucoma [4,40,90,91]. This aspect was indirectly observed by studying the VF in the early and moderate stages, in which the initial visual defect is twice more frequent in the superior hemifield than in the inferior one [60,92,93]. In the mild and moderate glaucoma stages, GCC and RNFL are similar in detecting the disease [39,91]. In these cases, the thinning of pRNFL and GCC thickness has a strong correlation with the severity of glaucoma. For this reason, GCC and pRNFL are excellent parameters for monitoring glaucoma. In these stages, OCT is the best tool for detecting the glaucoma changes that occur over time. Comparing GCC to pRNFL, Shin et al. observed a better correlation of GCC with the VF progression [47]. In moderate and advanced glaucoma, a statistically significant rate of progression for GCC (GCIPL) was correlated with the VF progression, while for pRNFL, such a correlation was not observed.

#### 7.1.2. The Value of GCC Compared to pRNFL for Myopia

OCT has limitations for the evaluation of pRNFL in patients with high myopia due to the aspect of the peripapillary retina. The GCC evaluation is a better diagnostic method than pRNFL for the detection of glaucoma in patients with high myopia [94,95]. In the study conducted by Soel et al., the GCC analysis proved to be the best parameter in detecting preperimetric glaucoma in myopic patients [90]. The eyes with high myopia have several changes compared to normal eyes, such as large and tilted disc, myopic temporal crescent and occasionally severe peripapillary staphyloma, which modify the thickness and the distribution of fibers around the optic disc.

#### 7.1.3. The Value of GCC Compared to pRNFL for Different Types of Glaucoma

Studies conducted on glaucoma patients with the same MD value of the VF have reported thinner pRNFL and GCC in subjects with PEX glaucoma than with POAG. The pattern of the GCC changes is different for PEX glaucoma, in which the defect is more severe and diffuse than in POAG for the similar MD value of the VF [96]. In another study, the GCC and RNFL were reported to be more reduced in patients with PEX glaucoma and PEX syndrome. The pattern of the macular thickness of the GCC and pRNFL loss is different for POAG, which means that the inferior part is thinner than the superior part compared to the other two entities [97]. However, another study observed that the superior part of the macular RNFL and GCIPL is thinner in the patients with PEX syndrome than in the normal ones [98]. The pattern of GCC loss is different in NTG versus POAG in contrast to pRNFL, which has the same changes in both types of glaucoma. NTG is characterized by a focal loss of GCC, while in POAG, the loss of GCC is rather diffuse [99]. In addition, in the early stage of POAG and NTG, the inferior arcuate region is the most vulnerable area, especially the distal portion of the inferior arcuate bundle which is greatly and most often affected by the disease. These findings agree with the GCC loss patterns stated in other studies that analyzed NTG in the Asian population [100].

### 7.2. The Comparison of GCC with the Cup/Disc Ratio

Several studies compared the cup/disc (c/d) ratio with RNFL and GCC. Patients with glaucoma show an increase of the c/d ratio, but the differences are not statistically significant in the early glaucoma vs. non-glaucomatous patients; therefore, the severity of glaucoma cannot be appreciated by using only this parameter. A study conducted by Lim S et al., published in 2020, assessed GCC, RNFL and the c/d ratio in patients with unilateral PEX glaucoma and compared the data of the affected and fellow eye with a control group (non-glaucomatous). They found statistical differences between the glaucomatous eye and the normal eye for GCC and RNFL, but no statistical difference for the c/d ratio [101]. Another study, which evaluated the c/d ratio, pRNFL and GCC, concluded that the c/d ratio and GCC are better tools than pRNFL for the evaluation of glaucomatous patients with high myopia [94].

### 7.3. Comparison of GCC with the Macular Thickness

Not all OCT devices can analyze the CGIPL. For this reason, some studies investigated the average values of the macular thickness and volume in glaucoma patients. They observed that both macular parameters decreased in glaucoma patients [88,102,103]. The reduction of the macular thickness and of the macular volume in glaucoma is basically due to the GCC loss. Several studies found a relation between the macular thickness and other structural and functional aspects of the retina, GCC, mRNFL and MD value of the VF [97,104,105]. The macular thickness varies with the axial length of the eye and with the sex of the subject [106]. Since the macular thickness variation is related to several individual parameters and to the thickness of the external layers, it has a limited role in the diagnosis of early glaucoma or in establishing the severity of glaucoma [88,103].

### 7.4. GCC and VF in the Glaucoma Patient

#### 7.4.1. Comparison of GCC with VF

The changes of GCC precede the changes of VF [107]. Several studies, which assess the glaucoma changes of GCC together with the functional changes evaluated by SAP (standard automated perimetry), confirm that the decrease of the GCC thickness precedes the decrease of the retina sensitivity detected on SAP [4,108]. For the glaucoma progression assessment, GCC is more reliable than VF in most stages of the disease. In the suspected and preperimetric glaucoma, GCC and pRNFL have better specificity and are superior to VF [109]. The VF event progression analysis is characterized by false positive errors (2.6%), which can lead to a “likely progression alert” and considerably low “possible progression alert” [110]. Additionally, in advanced stages, VF errors incline to false negative errors in areas of significant damage [111]. In advanced stages, pRNFL and, later, GCC achieve the floor effect. In this stage, it is more useful to evaluate the VF for monitoring the progression of glaucoma [43,112,113,114]. However, even though the VF is considered the best tool for evaluating the glaucoma, the recent study of Zhang et al. suggests that GCC is also very useful in evaluating advanced glaucoma [46,109]. In the advanced glaucoma, GCC continues to deplete when pRNFL achieves the minimum residual thickness. The difference is due to the regional aspect of pRNFL and GCC. For pRNFL, the evaluation of the nervous fiber layer focuses on the arcuate fibers, while GCC evaluates the central RGCs that are related to the papillomacular bundle. In early and moderate stages of glaucoma, the arcuate bundle is affected, while the macular bundle is relatively spared. In advanced glaucoma, the decrease of papillomacular bundle thickness is difficult to evaluate; conversely, GCC is easier to assess. In this stage, GCC continues to decrease and allows the evaluation of advanced glaucoma, together with VF.

#### 7.4.2. The Role of GCC and RNFL in the Prediction of VF Changes

Advanced images for glaucoma study (AIGS) concluded that the focal loss of GCC is a good predictor for the loss of VF. The prediction of the focal GCC loss is stronger than the focal loss of RNFL for cases of rapid VF loss. Zhang et al. defined the rapid loss of VF as a decrease of more than 1%/year of the VF index or MD slope < −0.5 dB/year, and the slow loss VF by a decrease of the VF index more than 0.5%/year or MD slope more than −0.25 dB/year [115]. The authors found that the best predictor for the rapid loss of VF is the focal loss of GCC. Other studies highlighted that the reduction of the GCC and of the pRNFL thickness is a good predictor for the VF loss in preperimetric or suspected glaucoma [108].

#### 7.4.3. Combining Indices for Glaucoma Disease Staging

Several studies combine structural and functional parameters for the glaucoma diagnosis. pRNFL is used for the structural aspect of glaucoma, while SAP is used for the functional one. These indices are empirical, and, because of this limitation, they are not currently used in the clinical practice [107,116,117].

## 8. Errors and Artefacts in the GCC Examination

SD-OCT has an important role in the diagnosis and treatment of glaucoma, but like any device, the acquired scans can have artefacts. Understanding the types of artefacts commonly seen in the imaging of patients who are evaluated for glaucoma will help the physicians to better interpret the data in order to achieve proper care for the patients. Studies have shown that pathologic ocular features are the most common cause of artefacts for macular thickness and RNFL scans [118]. Epiretinal membrane (ERM) represents the most common cause of artefacts in macular thickness scans, especially in RNFL scans. The software algorithm identifies the upper boundary of the ERM as that of the upper edge of the RNFL or as the internal limiting membrane of the retina, leading to measurement errors of the RNFL or of the macular thickness. An ERM can be identified by the presence of scalloped edges of a macular thickness map, and is the cause of an artificial increase of the central macular thickness. The presence of an ERM in a macular thickness scan should alert the physician regarding the possibility of an ERM in the RNFL scans [119]. The ERM can cause thinning or thickening of the macular GCIPL with irregular patterns. Thickening of GCIPL associated with ERM can lead to misidentification of the GCIPL thinning associated with glaucoma [120]. Furthermore, severe ERM can cause errors in the segmentation algorithm of the GCIPL thickness measurement. Another common ocular cause of artefact is the natural evolution of a posterior vitreous detachment, a stage in which the disease is associated with a traction on the inner limiting membrane. Areas of vitreous adhesion result in erroneously thickened RNFL measurements that subsequently decrease over time, with the release of the vitreous traction masquerading as progression of the RNFL thinning [119]. In addition to the ERM and the vitreous traction syndrome that have been previously discussed, other macular pathologies that can cause artefacts also are macular schisis and drusen. Myopic eyes due to a longer axial length are associated with a higher percentage of abnormal diagnostic classifications, because the RNFL normative databases are typically adjusted only by age and not by axial length or refractive error. Thinning of GCIPL associated with myopia is mainly located in the inferior or superior area, and presents as a horizontal crescent-shaped abnormal area [121,122]. Furthermore, myopic eyes are associated with many other artefacts, such as difficulty in acquiring a good image because of excessively long axial length or myopic retinal schisis affecting the peripapillary RNFL thickness.

Another artefact is related to the vitreomacular traction syndrome. As the fovea is an important landmark in automated retinal thickness assessment, an improperly centered fovea, secondary to vitreomacular traction, on the automated foveal localization (AFL) tool, may result in abnormal thickness map artefacts that lead to difficulties in managing pathology [123,124]. Hugh S. et al. investigated OCT scans of otherwise healthy eyes affected by vitreomacular traction, and observed that some Ganglion Cell Analysis maps had abnormal patterns that indicated eccentric thinning of the GCC. In such cases, the segmentation of the images is often false and there is an eccentric localization of AFL relative to the anatomical fovea, and the software displays an assumed pattern of the GCC thickness. That is, GCC is always absent at the site of AFL and increases in thickness radially outwards, with visual inspection of the scans often showing this segmentation to be false. When AFL determines the foveal center to be away from the true location, this pattern is projected onto the OCT at the site of the presumed foveal center, with a central GCC thickness of zero, despite its being visible on inspection [123,124]. Operator-dependent artefacts included truncation of the acquired SD-OCT image (i.e., all edges of the image were not within the acquisition window). This artefact results in erroneous mean measurements in the sector and in the global mean RNFL thickness values. A clue to identifying such artefacts is the presence of pRNFL values near zero, because even in the end-stage glaucoma, the pRNFL thickness remains approximately 30 µm due to a floor effect of the glial cell thickness [125]. Incorrect RNFL circle placement is another operator-dependent artefact. Although most of these artefacts have been reported to be mild, moderate to severe displacement of the circle may result in erroneous RNFL values. In some SD-OCT instruments (e.g., Cirrus), the points along a peripapillary concentric circle are identified by the software, eliminating this type of artefact [126].

The SD-OCT software-related artefacts included the misidentification of the retinal boundaries. This is commonly seen in eyes with high myopia, eyes with prominent posterior hyaloid or eyes with significant media opacities, because of poor image quality. Previous studies of the application of SD-OCT to retinal pathologic features have disclosed multiple sources of error that dramatically decrease the accuracy of these macular thickness measurements [127,128]. The most obvious source of error may be imprecise retinal layer segmentation, which can result from poor signal quality of the SD-OCT image or outright failure of the segmentation algorithm in otherwise high-quality images. Poor signal strength has been demonstrated as a major source of artefacts in other studies as well, precluding the ability to detect change in pRNFL over time [129,130]. One sign of inaccurate inner layer segmentation is the appearance of nonpathological shapes in the thickness and probability maps, such as a corner of abnormal thinning. Errors in the GCL-IPL segmentation can also often appear as segments of blue (thinning) on the thickness map in the shape of spokes of a wheel. A GCIPL reading of less than 40 μm is also typically indicative of areas of segmentation error. On the B-scan, the algorithm’s identification of the boundaries of the GCL and IPL often collapse together in the areas of artefact, producing an artefactual thinning [131]. Fitzgerald J et al. performed a study to determine the frequency of scan artefacts and errors in GCIPL imaging in individuals undergoing HD-OCT surveillance for glaucoma [132]. It was noted that 6.0% of all scans had either acquisition errors, segmentation artefacts or other macular pathologies. Acquisition errors can also be due to poor alignment by the operator. Part of the macula is anterior to the volume imaged by the macular cube. This results in missing data from the Ganglion Cell Analysis. Other artefacts were caused by machine segmentation errors, which can be readily evident as a focal, radial wedge of thinning on the macular GCIPL thickness map, without obvious pathology on the OCT B-scan. The presence of vitreous floaters can focally obscure the signal from the retina, leading to the inability to segment the retinal layers. The effect on the scan will depend on the location of the floater at the time of the scan. If the floater is outside the elliptical annulus scan area, it has a negligible effect on the segmentation.

## 9. Conclusions

As previously presented, there are many correlations between the changes in GCC and the RNFL in various neurological and ophthalmological diseases that involve the optic nerve, including glaucoma. For glaucoma evaluation, in the early stages, GCC analysis is the most sensitive examination and statistically significantly better than pRNFL. In the mild and moderate glaucoma stages, GCC and RNFL are similar in detecting the disease, with the thinning thickness of pRNFL and GCC having a strong correlation with the severity of glaucoma. For this reason, in these stages, GCC and pRNFL are excellent parameters for monitoring glaucoma and therefore, OCT is the best tool for detecting the glaucoma changes that occur over time. In advanced glaucoma, VF is considered the best tool for evaluating the glaucoma due to the floor effect achieved by the OCT parameters in this stage. In addition, the loss of GCC is a good predictor for the loss of VF and the prediction is stronger compared to the loss of RNFL. Eventually, OCT is an important tool in assessing glaucoma or other diseases that affect the optic nerve, with the mention that the correlations between GCC and RNFL have superior efficacy when monitoring the progression of glaucoma, as well as for differentiating between other diseases that can affect the optic nerve.

## Figures and Tables

**Table 1:** Summary of the GCC, RNFL and VF parameters in different glaucoma stages.

Parameter	Early-Stage Glaucoma	Moderate-Stage Glaucoma	Advanced-Stage Glaucoma
GCC	Inferior and inferotemporal quadrant thickness are the most sensitive parameters	Same as early-stage glaucoma	Superior quadrant thickness best correlated to MD value
RNFL	Inferior quadrant thickness is a sensitive parameter	Inferior quadrant thickness has a stronger correlation to MD value on VF	Not very reliable (reaches the floor effect before GCC)
VF (MD value)	Less sensitive than RNFL and GCC	Some studies show correlation with parameters of the RNFL and GCC	Superior RNFL and slightly superior or similar with GCC

**Table 2:** Summary of the OCT criteria for early-stage glaucoma diagnosis.

Parameter	OCT Aspects in the Early Glaucoma	Observation
GCC	Loss of the thickness in the inferotemporal quadrant (sometimes the loss is outside the examination area)	Related to MVZ
RNFL	Loss of the thickness in the inferior quadrant	Appears after GCC thickness loss in the inferotemporal quadrant
Inferior-superior asymmetry	Asymmetry of superior and inferior sectors of the temporal quadrant of the GCC	Appears after focal loss of GCC
Interocular GCC asymmetry	Global and inferior segment asymmetry of the GCC	GCC intraocular asymmetry is associated with RNFL and ONH asymmetry Exception is PEX syndrome

## Data Availability

Not applicable.

## References

[1] Devalla S.K., Liang Z., Pham T.H., Boote C., Strouthidis N.G., Thiery A.H., Girard M.J.A. (2020). Glaucoma Management in the Era of Artificial Intelligence. Br. J. Ophthalmol..

[2] Tatham A.J., Medeiros F.A. (2017). Detecting Structural Progression in Glaucoma with Optical Coherence Tomography. Ophthalmology.

[3] Renard J.-P., Fénolland J.-R., Giraud J.-M. (2019). Glaucoma Progression Analysis by Spectral-Domain Optical Coherence Tomography (SD-OCT). J. Fr. Ophtalmol..

[4] Scuderi G., Fragiotta S., Scuderi L., Iodice C.M., Perdicchi A. (2020). Ganglion Cell Complex Analysis in Glaucoma Patients: What Can It Tell Us?. Eye Brain.

[5] Nouri-Mahdavi K., Nowroozizadeh S., Nassiri N., Cirineo N., Knipping S., Giaconi J., Caprioli J. (2013). Macular Ganglion Cell/Inner Plexiform Layer Measurements by Spectral Domain Optical Coherence Tomography for Detection of Early Glaucoma and Comparison to Retinal Nerve Fiber Layer Measurements. Am. J. Ophthalmol..

[6] Koh V., Tham Y.-C., Cheung C.Y., Mani B., Wong T.Y., Aung T., Cheng C.-Y. (2018). Diagnostic Accuracy of Macular Ganglion Cell-Inner Plexiform Layer Thickness for Glaucoma Detection in a Population-Based Study: Comparison with Optic Nerve Head Imaging Parameters. PLoS ONE.

[7] Curcio C.A., Allen K.A. (1990). Topography of Ganglion Cells in Human Retina. J. Comp. Neurol..

[8] Kansal V., Armstrong J.J., Pintwala R., Hutnik C. (2018). Optical Coherence Tomography for Glaucoma Diagnosis: An Evidence Based Meta-Analysis. PLoS ONE.

[9] Tan O., Chopra V., Lu A.T.-H., Schuman J.S., Ishikawa H., Wollstein G., Varma R., Huang D. (2009). Detection of Macular Ganglion Cell Loss in Glaucoma by Fourier-Domain Optical Coherence Tomography. Ophthalmology.

[10] Takagi S.T., Kita Y., Yagi F., Tomita G. (2012). Macular Retinal Ganglion Cell Complex Damage in the Apparently Normal Visual Field of Glaucomatous Eyes With Hemifield Defects. J. Glaucoma.

[11] Lisboa R., Paranhos A., Weinreb R.N., Zangwill L.M., Leite M.T., Medeiros F.A. (2013). Comparison of Different Spectral Domain OCT Scanning Protocols for Diagnosing Preperimetric Glaucoma. Invest. Ophthalmol. Vis. Sci..

[12] Feng L., Zhao Y., Yoshida M., Chen H., Yang J.F., Kim T.S., Cang J., Troy J.B., Liu X. (2013). Sustained Ocular Hypertension Induces Dendritic Degeneration of Mouse Retinal Ganglion Cells That Depends on Cell Type and Location. Investig. Ophthalmol. Vis. Sci..

[13] Dascalescu D., Corbu C., Coviltir V., Schmitzer S., Constantin M., Burcel M., Ionescu C., Strehaianu V., Potop V. (2018). The Ganglion Cell Complex as an Useful Tool in Glaucoma Assessment. Rom J. Ophthalmol..

[14] Cennamo G., Romano M.R., Vecchio E.C., Minervino C., Della Guardia C., Velotti N., Carotenuto A., Montella S., Orefice G., Cennamo G. (2016). Anatomical and Functional Retinal Changes in Multiple Sclerosis. Eye (Lond).

[15] Pierro L., Rabiolo A. (2017). Emerging Issues for Optical Coherence Tomography. Dev. Ophthalmol. Karger Basel.

[16] Wang B., Camino A., Pi S., Guo Y., Wang J., Huang D., Hwang T.S., Jia Y. (2019). Three-Dimensional Structural and Angiographic Evaluation of Foveal Ischemia in Diabetic Retinopathy: Method and Validation. Biomed. Opt. Express.

[17] Glovinsky Y., Quigley H.A., Pease M.E. (1993). Foveal Ganglion Cell Loss Is Size Dependent in Experimental Glaucoma. Investig. Ophthalmol. Vis. Sci..

[18] Murthy R.K., Haji S., Sambhav K., Grover S., Chalam K.V. (2016). Clinical Applications of Spectral Domain Optical Coherence Tomography in Retinal Diseases. Biomed. J..

[19] Verticchio Vercellin A.C., Jassim F., Poon L.Y.-C., Tsikata E., Braaf B., Shah S., Ben-David G., Shieh E., Lee R., Simavli H. (2018). Diagnostic Capability of Three-Dimensional Macular Parameters for Glaucoma Using Optical Coherence Tomography Volume Scans. Investig. Ophthalmol. Vis. Sci..

[20] Mwanza J.-C., Oakley J.D., Budenz D.L., Chang R.T., Knight O.J., Feuer W.J. (2011). Macular Ganglion Cell-Inner Plexiform Layer: Automated Detection and Thickness Reproducibility with Spectral Domain-Optical Coherence Tomography in Glaucoma. Investig. Ophthalmol. Vis. Sci..

[21] Pazos M., Dyrda A.A., Biarnés M., Gómez A., Martín C., Mora C., Fatti G., Antón A. (2017). Diagnostic Accuracy of Spectralis SD OCT Automated Macular Layers Segmentation to Discriminate Normal from Early Glaucomatous Eyes. Ophthalmology.

[22] Akashi A., Kanamori A., Nakamura M., Fujihara M., Yamada Y., Negi A. (2013). Comparative Assessment for the Ability of Cirrus, RTVue, and 3D-OCT to Diagnose Glaucoma. Investig. Ophthalmol. Vis. Sci..

[23] Kita Y., Soutome N., Horie D., Kita R., Hollό G. (2017). Circumpapillary Ganglion Cell Complex Thickness to Diagnose Glaucoma: A Pilot Study. Indian J. Ophthalmol..

[24] Chaglasian M., Fingeret M., Davey P.G., Huang W.-C., Leung D., Ng E., Reisman C.A. (2018). The Development of a Reference Database with the Topcon 3D OCT-1 Maestro. Clin. Ophthalmol..

[25] Donaldson L., Margolin E. (2021). Visual Fields and Optical Coherence Tomography (OCT) in Neuro-Ophthalmology: Structure-Function Correlation. J. Neurol. Sci..

[26] Petzold A. (2021). Three “Red Lines” for Pattern Recognition-Based Differential Diagnosis Using Optical Coherence Tomography in Clinical Practice. J. Neuroophthalmol..

[27] Jørstad Ø.K., Wigers A.R., Marthinsen P.B., Moe M.C., Evang J.A. (2018). Loss of Horizontal Macular Ganglion Cell Complex Asymmetry: An Optical Coherence Tomography Indicator of Chiasmal Compression. BMJ Open Ophthalmol..

[28] Tieger M.G., Hedges T.R., Ho J., Erlich-Malona N.K., Vuong L.N., Athappilly G.K., Mendoza-Santiesteban C.E. (2017). Ganglion Cell Complex Loss in Chiasmal Compression by Brain Tumors. J. Neuro-Ophthalmol..

[29] Goto K., Miki A., Yamashita T., Araki S., Takizawa G., Mizukawa K., Ieki Y., Kiryu J. (2017). Quantitative Analysis of Macular Inner Retinal Layer Using Swept-Source Optical Coherence Tomography in Patients with Optic Tract Syndrome. J. Ophthalmol..

[30] Yamashita T., Miki A., Goto K., Araki S., Takizawa G., Ieki Y., Kiryu J., Tabuchi A., Iguchi Y., Kimura K. (2020). Evaluation of Significance Maps and the Analysis of the Longitudinal Time Course of the Macular Ganglion Cell Complex Thicknesses in Acquired Occipital Homonymous Hemianopia Using Spectral-Domain Optical Coherence Tomography. Neuro-Ophthalmol..

[31] Wang X., Li S., Fu J., Wu G., Mu D., Li S., Wang J., Wang N. (2011). Comparative Study of Retinal Nerve Fibre Layer Measurement by RTVue OCT and GDx VCC. Br. J. Ophthalmol..

[32] Leite M.T., Rao H.L., Zangwill L.M., Weinreb R.N., Medeiros F.A. (2011). Comparison of the Diagnostic Accuracies of the Spectralis, Cirrus, and RTVue Optical Coherence Tomography Devices in Glaucoma. Ophthalmology.

[33] Rao H.L., Zangwill L.M., Weinreb R.N., Sample P.A., Alencar L.M., Medeiros F.A. (2010). Comparison of Different Spectral Domain Optical Coherence Tomography Scanning Areas for Glaucoma Diagnosis. Ophthalmology.

[34] Park S.B., Sung K.R., Kang S.Y., Kim K.R., Kook M.S. (2009). Comparison of Glaucoma Diagnostic Capabilities of Cirrus HD and Stratus Optical Coherence Tomography. Arch. Ophthalmol..

[35] Moreno P.A.M., Konno B., Lima V.C., Castro D.P.E., Castro L.C., Leite M.T., Mendes Pacheco M.A.M., Lee J.M., Prata T.S. (2011). Spectral-Domain Optical Coherence Tomography for Early Glaucoma Assessment: Analysis of Macular Ganglion Cell Complex versus Peripapillary Retinal Nerve Fiber Layer. Can. J. Ophthalmol..

[36] Traverso C.E., Walt J.G., Kelly S.P., Hommer A.H., Bron A.M., Denis P., Nordmann J.P., Renard J.P., Bayer A., Grehn F. (2005). Direct Costs of Glaucoma and Severity of the Disease: A Multinational Long Term Study of Resource Utilisation in Europe. Br. J. Ophthalmol..

[37] Choi J.A., Shin H.-Y., Park H.-Y.L., Park C.K. (2017). The Pattern of Retinal Nerve Fiber Layer and Macular Ganglion Cell-Inner Plexiform Layer Thickness Changes in Glaucoma. J. Ophthalmol..

[38] Miraftabi A., Amini N., Morales E., Henry S., Yu F., Afifi A., Coleman A.L., Caprioli J., Nouri-Mahdavi K. (2016). Macular SD-OCT Outcome Measures: Comparison of Local Structure-Function Relationships and Dynamic Range. Investig. Opthalmology Vis. Sci..

[39] Kim N.R., Lee E.S., Seong G.J., Kim J.H., An H.G., Kim C.Y. (2010). Structure–Function Relationship and Diagnostic Value of Macular Ganglion Cell Complex Measurement Using Fourier-Domain OCT in Glaucoma. Investig. Opthalmology Vis. Sci..

[40] Rao H.L., Qasim M., Hussain R.S.M., Januwada M., Pillutla L.N., Begum V.U., Chaitanya A., Senthil S., Garudadri C.S. (2015). Structure–Function Relationship in Glaucoma Using Ganglion Cell–Inner Plexiform Layer Thickness Measurements. Investig. Opthalmology Vis. Sci..

[41] Moghimi S., Bowd C., Zangwill L.M., Penteado R.C., Hasenstab K., Hou H., Ghahari E., Manalastas P.I.C., Proudfoot J., Weinreb R.N. (2019). Measurement Floors and Dynamic Ranges of OCT and OCT Angiography in Glaucoma. Ophthalmology.

[42] Mwanza J.-C., Budenz D.L., Warren J.L., Webel A.D., Reynolds C.E., Barbosa D.T., Lin S. (2015). Retinal Nerve Fibre Layer Thickness Floor and Corresponding Functional Loss in Glaucoma. Br. J. Ophthalmol..

[43] Hood D.C., Kardon R.H. (2007). A Framework for Comparing Structural and Functional Measures of Glaucomatous Damage. Prog. Retin. Eye Res..

[44] Hood D.C., Anderson S.C., Wall M., Kardon R.H. (2007). Structure versus Function in Glaucoma: An Application of a Linear Model. Investig. Opthalmology Vis. Sci..

[45] Bowd C., Zangwill L.M., Weinreb R.N., Medeiros F.A., Belghith A. (2017). Estimating Optical Coherence Tomography Structural Measurement Floors to Improve Detection of Progression in Advanced Glaucoma. Am. J. Ophthalmol..

[46] Sung K.R., Sun J.H., Na J.H., Lee J.Y., Lee Y. (2012). Progression Detection Capability of Macular Thickness in Advanced Glaucomatous Eyes. Ophthalmology.

[47] Shin J.W., Sung K.R., Lee G.C., Durbin M.K., Cheng D. (2017). Ganglion Cell–Inner Plexiform Layer Change Detected by Optical Coherence Tomography Indicates Progression in Advanced Glaucoma. Ophthalmology.

[48] Zhang X., Dastiridou A., Francis B.A., Tan O., Varma R., Greenfield D.S., Schuman J.S., Sehi M., Chopra V., Huang D. (2016). Baseline Fourier-Domain Optical Coherence Tomography Structural Risk Factors for Visual Field Progression in the Advanced Imaging for Glaucoma Study. Am. J. Ophthalmol..

[49] Lee W.J., Baek S.U., Kim Y.K., Park K.H., Jeoung J.W. (2019). Rates of Ganglion Cell-Inner Plexiform Layer Thinning in Normal, Open-Angle Glaucoma and Pseudoexfoliation Glaucoma Eyes: A Trend-Based Analysis. Investig. Ophthalmol. Vis. Sci..

[50] Lee W.J., Kim Y.K., Park K.H., Jeoung J.W. (2017). Trend-Based Analysis of Ganglion Cell–Inner Plexiform Layer Thickness Changes on Optical Coherence Tomography in Glaucoma Progression. Ophthalmology.

[51] Leung C.K.S., Ye C., Weinreb R.N., Yu M., Lai G., Lam D.S. (2013). Impact of Age-Related Change of Retinal Nerve Fiber Layer and Macular Thicknesses on Evaluation of Glaucoma Progression. Ophthalmology.

[52] Holló G., Zhou Q. (2016). Evaluation of Retinal Nerve Fiber Layer Thickness and Ganglion Cell Complex Progression Rates in Healthy, Ocular Hypertensive, and Glaucoma Eyes with the Avanti RTVue-XR Optical Coherence Tomograph Based on 5-Year Follow-Up. J. Glaucoma.

[53] Hood D.C., Raza A.S., de Moraes C.G.V., Liebmann J.M., Ritch R. (2013). Glaucomatous Damage of the Macula. Prog. Retin. Eye Res..

[54] Huang G., Luo T., Gast T.J., Burns S.A., Malinovsky V.E., Swanson W.H. (2015). Imaging Glaucomatous Damage Across the Temporal Raphe. Investig. Opthalmology Vis. Sci..

[55] Huang G., Gast T.J., Burns S.A. (2014). In Vivo Adaptive Optics Imaging of the Temporal Raphe and Its Relationship to the Optic Disc and Fovea in the Human Retina. Investig. Opthalmology Vis. Sci..

[56] Swanson W.H., King B.J., Burns S.A. (2019). Within-Subject Variability in Human Retinal Nerve Fiber Bundle Width. PLoS ONE.

[57] Hood D.C., Raza A.S., de Moraes C.G.v, Johnson C.A., Liebmann J.M., Ritch R. (2012). The Nature of Macular Damage in Glaucoma as Revealed by Averaging Optical Coherence Tomography Data. Transl. Vis. Sci. Technol..

[58] Jonas J.B., Fernández M.C., Stürmer J. (1993). Pattern of Glaucomatous Neuroretinal Rim Loss. Ophthalmology.

[59] Jonas J.B., Mardin C.Y., Schlötzer-Schrehardt U., Naumann G.O. (1991). Morphometry of the Human Lamina Cribrosa Surface. Investig. Ophthalmol. Vis. Sci..

[60] Choi J.A., Park H.-Y.L., Jung K.-I., Hong K.H., Park C.K. (2013). Difference in the Properties of Retinal Nerve Fiber Layer Defect Between Superior and Inferior Visual Field Loss in Glaucoma. Investig. Opthalmology Vis. Sci..

[61] Heijl A., Lundqvist L. (2009). The frequency distribution of earliest glaucomatous visual field defects documented by automatic perimetry. Acta Ophthalmol.

[62] Tanna A.P. (2020). Retinal Nerve Fiber Layer Analysis in Glaucoma. Atlas of Optical Coherence Tomography for Glaucoma.

[63] Quigley H.A., Addicks E.M. (1981). Regional Differences in the Structure of the Lamina Cribrosa and Their Relation to Glaucomatous Optic Nerve Damage. Arch. Ophthalmol..

[64] Dandona L., Quigley H.A., Brown A.E., Enger C. (1990). Quantitative Regional Structure of the Normal Human Lamina Cribrosa: A Racial Comparison. Arch. Ophthalmol..

[65] Kotowski J., Folio L.S., Wollstein G., Ishikawa H., Ling Y., Bilonick R.A., Kagemann L., Schuman J.S. (2012). Glaucoma Discrimination of Segmented Cirrus Spectral Domain Optical Coherence Tomography (SD-OCT) Macular Scans. Br. J. Ophthalmol..

[66] Takemoto D., Higashide T., Ohkubo S., Udagawa S., Sugiyama K. (2020). Ability of Macular Inner Retinal Layer Thickness Asymmetry Evaluated by Optical Coherence Tomography to Detect Preperimetric Glaucoma. Transl. Vis. Sci. Technol..

[67] Yamada H., Hangai M., Nakano N., Takayama K., Kimura Y., Miyake M., Akagi T., Ikeda H.O., Noma H., Yoshimura N. (2014). Asymmetry Analysis of Macular Inner Retinal Layers for Glaucoma Diagnosis. Am. J. Ophthalmol..

[68] Kim Y.K., Yoo B.W., Kim H.C., Park K.H. (2015). Automated Detection of Hemifield Difference across Horizontal Raphe on Ganglion Cell-Inner Plexiform Layer Thickness Map. Ophthalmology.

[69] Lee S.Y., Lee E.K., Park K.H., Kim D.M., Jeoung J.W. (2016). Asymmetry Analysis of Macular Inner Retinal Layers for Glaucoma Diagnosis: Swept-Source Optical Coherence Tomography Study. PLoS ONE.

[70] Asrani S. (2011). Novel Software Strategy for Glaucoma Diagnosis. Arch. Ophthalmol..

[71] Mwanza J.C., Durbin M.K., Budenz D.L. (2011). Interocular Symmetry in Peripapillary Retinal Nerve Fiber Layer Thickness Measured with the Cirrus HD-OCT in Healthy Eyes. Am. J. Ophthalmol..

[72] Choi Y.J., Jeoung J.W., Park K.H., Kim D.M. (2013). Glaucoma Detection Ability of Ganglion Cell-Inner Plexiform Layer Thickness by Spectral-Domain Optical Coherence Tomography in High Myopia. Investig. Ophthalmol. Vis. Sci..

[73] Jeoung J.W., Choi Y.J., Park K.H., Kim D.M. (2013). Macular Ganglion Cell Imaging Study: Glaucoma Diagnostic Accuracy of Spectral-Domain Optical Coherence Tomography. Investig. Ophthalmol. Vis. Sci..

[74] Takayama K., Hangai M., Durbin M., Nakano N., Morooka S., Akagi T., Ikeda H.O., Yoshimura N. (2012). A Novel Method to Detect Local Ganglion Cell Loss in Early Glaucoma Using Spectral-Domain Optical Coherence Tomography. Investig. Ophthalmol. Vis. Sci..

[75] Lee S.Y., Jeoung J.W., Park K.H., Kim D.M. (2015). Macular Ganglion Cell Imaging Study: Interocular Symmetry of Ganglion Cell-Inner Plexiform Layer Thickness in Normal Healthy Eyes. Am. J. Ophthalmol..

[76] Sullivan-Mee M., Ruegg C.C., Pensyl D., Halverson K., Qualls C. (2013). Diagnostic Precision of Retinal Nerve Fiber Layer and Macular Thickness Asymmetry Parameters for Identifying Early Primary Open-Angle Glaucoma. Am. J. Ophthalmol..

[77] Williams A.L., Gatla S., Leiby B.E., Fahmy I., Biswas A., De Barros D.M., Ramakrishnan R., Bhardwaj S., Wright C., Dubey S. (2013). The Value of Intraocular Pressure Asymmetry in Diagnosing Glaucoma. J. Glaucoma.

[78] Li H., Healey P.R., Tariq Y.M., Teber E., Mitchell P. (2013). Symmetry of Optic Nerve Head Parameters Measured by the Heidelberg Retina Tomograph 3 in Healthy Eyes: The Blue Mountains Eye Study. Am. J. Ophthalmol..

[79] Field M.G., Alasil T., Baniasadi N., Que C., Simavli H., Sobeih D., Sola-Del Valle D., Best M.J., Chen T.C. (2016). Facilitating Glaucoma Diagnosis with Intereye Retinal Nerve Fiber Layer Asymmetry Using Spectral-Domain Optical Coherence Tomography. J. Glaucoma.

[80] Iester M., Telani S., Perdicchi A., Manni G.L., Uva M., Figus M., Perdicchi A., Brusini P., Paoli D., Rolando M. (2012). Differences in Central Corneal Thickness between the Paired Eyes and the Severity of the Glaucomatous Damage. Eye.

[81] Levine R.A., Demirel S., Fan J., Keltner J.L., Johnson C.A., Kass M.A., Budenz D.L., Fantes F.E., Gedde S.J., Parrish R.K. (2006). Asymmetries and Visual Field Summaries as Predictors of Glaucoma in the Ocular Hypertension Treatment Study. Investig. Ophthalmol. Vis. Sci..

[82] Aydin D., Kusbeci T., Uzunel U.D., Orsel T., Yuksel B. (2016). Evaluation of Retinal Nerve Fiber Layer and Ganglion Cell Complex Thickness in Unilateral Exfoliation Syndrome Using Optical Coherence Tomography. J. Glaucoma.

[83] Hammer T., Schlötzer-Schrehardt U., Naumann G.O.H. (2001). Unilateral or Asymmetric Pseudoexfoliation Syndrome?. Arch. Ophthalmol..

[84] Xu H., Zong Y., Zhai R., Kong X., Jiang C., Sun X. (2019). Intereye and Intraeye Asymmetry Analysis of Retinal Microvascular and Neural Structure Parameters for Diagnosis of Primary Open-Angle Glaucoma. Eye.

[85] Rao H.L., Pradhan Z.S., Weinreb R.N., Reddy H.B., Riyazuddin M., Dasari S., Palakurthy M., Puttaiah N.K., Rao D.A.S., Webers C.A.B. (2016). Regional Comparisons of Optical Coherence Tomography Angiography Vessel Density in Primary Open-Angle Glaucoma. Am. J. Ophthalmol..

[86] Le P.v., Tan O., Chopra V., Francis B.A., Ragab O., Varma R., Huang D. (2013). Regional Correlation Among Ganglion Cell Complex, Nerve Fiber Layer, and Visual Field Loss in Glaucoma. Investig. Opthalmology Vis. Sci..

[87] Barua N., Sitaraman C., Goel S., Chakraborti C., Mukherjee S., Parashar H. (2016). Comparison of Diagnostic Capability of Macular Ganglion Cell Complex and Retinal Nerve Fiber Layer among Primary Open Angle Glaucoma, Ocular Hypertension, and Normal Population Using Fourier-Domain Optical Coherence Tomography and Determining Their Functional Correlation in Indian Population. Indian J. Ophthalmol..

[88] Oli A., Joshi D. (2015). Can Ganglion Cell Complex Assessment on Cirrus HD OCT Aid in Detection of Early Glaucoma?. Saudi J. Ophthalmol..

[89] Cennamo G., Montorio D., Romano M.R., Cardone D.M., Minervino C., Reibaldi M., Cennamo G. (2016). Structure-Functional Parameters in Differentiating Between Patients With Different Degrees of Glaucoma. J. Glaucoma.

[90] Seol B.R., Jeoung J.W., Park K.H. (2015). Glaucoma Detection Ability of Macular Ganglion Cell-Inner Plexiform Layer Thickness in Myopic Preperimetric Glaucoma. Investig. Opthalmology Vis. Sci..

[91] Hong S.W., Lee S.B., Jee D., Ahn M.D. (2016). Evaluation of Interocular Retinal Nerve Fiber Layer Thickness Symmetry as a Diagnostic Modality for Glaucoma. J. Glaucoma.

[92] Drance S.M. (1977). The Visual Field of Low Tension Glaucoma and Shock-Induced Optic Neuropathy. Arch. Ophthalmol..

[93] Nicholas S.P., Werner E.B. (1980). Location of Early Glaucomatous Visual Field Defects. Can. J. Ophthalmol..

[94] Zhang Y., Wen W., Sun X. (2016). Comparison of Several Parameters in Two Optical Coherence Tomography Systems for Detecting Glaucomatous Defects in High Myopia. Investig. Opthalmology Vis. Sci..

[95] Shoji T., Sato H., Ishida M., Takeuchi M., Chihara E. (2011). Assessment of Glaucomatous Changes in Subjects with High Myopia Using Spectral Domain Optical Coherence Tomography. Investig. Opthalmology Vis. Sci..

[96] Aydin D. (2017). Comparison of Ganglion Cell Complex Defect Patterns of Exfoliative Glaucoma and Prymary Open Angle Glaucoma Using Optycal Coherence Tomography. JOJ Ophthalmol..

[97] Saricaoglu M.S., Karakurt A., Hamurcu M., Ekicier Acar S. (2017). Comparison of the Ganglion Cell Complex and Retinal Nerve Fiber Layer Thickness in Pseudoexfoliation Syndrome, Pseudoexfoliation Glaucoma and Healthy Subjects. New Front. Ophthalmol..

[98] Eltutar K., Acar F., Kayaarası Öztürker Z., Ünsal E., Özdoğan Erkul S. (2015). Structural Changes in Pseudoexfoliation Syndrome Evaluated with Spectral Domain Optical Coherence Tomography. Curr. Eye Res..

[99] Kim N.R., Hong S., Kim J.H., Rho S.S., Seong G.J., Kim C.Y. (2013). Comparison of Macular Ganglion Cell Complex Thickness by Fourier-Domain OCT in Normal Tension Glaucoma and Primary Open-Angle Glaucoma. J. Glaucoma.

[100] Omodaka K., Takada N., Takahashi H., Nakazawa T. (2015). Regional Structural Vulnerability of the Macula in Patients with Normal Tension Glaucoma. Clin. Exp. Ophthalmol..

[101] Lim S.-H., Gu W.M., Cha S.C. (2020). Comparison of the Retinal Nerve Fiber Layer and Ganglion Cell Complex Thickness in Korean Patients with Unilateral Exfoliation Syndrome and Healthy Subjects. Eye.

[102] Barisić F., Sicaja A.J., Ravlić M.M., Novak-Laus K., Iveković R., Mandić Z. (2012). Macular Thickness and Volume Parameters Measured Using Optical Coherence Tomography (OCT) for Evaluation of Glaucoma Patients. Coll. Antropol..

[103] Sathyan P., Agarwal P., Sharma A., Saini V. (2014). Macular Thickness Variability in Primary Open Angle Glaucoma Patients Using Optical Coherence Tomography. J. Curr. Glaucoma Pr..

[104] Greenfield D.S. (2003). Macular Thickness Changes in Glaucomatous Optic Neuropathy Detected Using Optical Coherence Tomography. Arch. Ophthalmol..

[105] Leung C.K.S., Chan W.-M., Yung W.-H., Ng A.C.K., Woo J., Tsang M.-K., Tse R.K.K. (2005). Comparison of Macular and Peripapillary Measurements for the Detection of Glaucoma. Ophthalmology.

[106] Pokharel A., Shrestha J.B., Shrestha G.S. (2016). Macular Thickness and Macular Volume Measurements Using Spectral Domain Optical Coherence Tomography in Normal Nepalese Eyes. Clin. Ophthalmol..

[107] Medeiros F.A., Lisboa R., Weinreb R.N., Girkin C.A., Liebmann J.M., Zangwill L.M. (2012). A Combined Index of Structure and Function for Staging Glaucomatous Damage. Arch. Ophthalmol..

[108] Zhang X., Loewen N., Tan O., Greenfield D.S., Schuman J.S., Varma R., Huang D., Huang D., Francis B., Greenfield D.S. (2016). Predicting Development of Glaucomatous Visual Field Conversion Using Baseline Fourier-Domain Optical Coherence Tomography. Am. J. Ophthalmol..

[109] Zhang X., Dastiridou A., Francis B.A., Tan O., Varma R., Greenfield D.S., Schuman J.S., Huang D. (2017). Comparison of Glaucoma Progression Detection by Optical Coherence Tomography and Visual Field. Am. J. Ophthalmol..

[110] Artes P.H., O’Leary N., Nicolela M.T., Chauhan B.C., Crabb D.P. (2014). Visual Field Progression in Glaucoma. Ophthalmology.

[111] Saunders L.J., Russell R.A., Crabb D.P. (2015). Measurement Precision in a Series of Visual Fields Acquired by the Standard and Fast Versions of the Swedish Interactive Thresholding Algorithm. JAMA Ophthalmol..

[112] Abe R.Y., Diniz-Filho A., Zangwill L.M., Gracitelli C.P.B., Marvasti A.H., Weinreb R.N., Baig S., Medeiros F.A. (2016). The Relative Odds of Progressing by Structural and Functional Tests in Glaucoma. Investig. Opthalmology Vis. Sci..

[113] Sommer A. (1991). Clinically Detectable Nerve Fiber Atrophy Precedes the Onset of Glaucomatous Field Loss. Arch. Ophthalmol..

[114] Banegas S.A., Antón A., Morilla A., Bogado M., Ayala E.M., Fernandez-Guardiola A., Moreno-Montañes J. (2016). Evaluation of the Retinal Nerve Fiber Layer Thickness, the Mean Deviation, and the Visual Field Index in Progressive Glaucoma. J. Glaucoma.

[115] Zhang X., Parrish R.K., Greenfield D.S., Francis B.A., Varma R., Schuman J.S., Tan O., Huang D. (2019). Predictive Factors for the Rate of Visual Field Progression in the Advanced Imaging for Glaucoma Study. Am. J. Ophthalmol..

[116] Distante P., Lombardo S., Verticchio Vercellin A.C., Raimondi M., Rolando M., Tinelli C., Milano G. (2015). Structure/Function Relationship and Retinal Ganglion Cells Counts to Discriminate Glaucomatous Damages. BMC Ophthalmol..

[117] Wu Z., Medeiros F.A. (2021). A Simplified Combined Index of Structure and Function for Detecting and Staging Glaucomatous Damage. Sci. Rep..

[118] Han I.C., Jaffe G.J. (2010). Evaluation of Artifacts Associated with Macular Spectral-Domain Optical Coherence Tomography. Ophthalmology.

[119] Giani A., Cigada M., Esmaili D.D., Salvetti P., Luccarelli S., Marziani E., Luiselli C., Sabella P., Cereda M., Eandi C. (2010). Artifacts in Automatic Retinal Segmentation Using Different Optical Coherence Tomography Instruments. Retina.

[120] Hwang Y.H. (2014). Patterns of Macular Ganglion Cell Abnormalities in Various Ocular Conditions. Investig. Opthalmology Vis. Sci..

[121] Yoo Y.C., Lee C.M., Park J.H. (2012). Changes in Peripapillary Retinal Nerve Fiber Layer Distribution by Axial Length. Optom. Vis. Sci..

[122] Qiu K.L., Zhang M.Z., Leung C.K.-S., Zhang R.P., Lu X.H., Wang G., Lam D.S.C. (2011). Diagnostic Classification of Retinal Nerve Fiber Layer Measurement in Myopic Eyes: A Comparison Between Time-Domain and Spectral-Domain Optical Coherence Tomography. Am. J. Ophthalmol..

[123] Liefers B., Venhuizen F.G., Schreur V., van Ginneken B., Hoyng C., Fauser S., Theelen T., Sánchez C.I. (2017). Automatic Detection of the Foveal Center in Optical Coherence Tomography. Biomed. Opt. Express.

[124] Gregori G., Knighton R.W., Puliafito C.A., Legarreta J.E., Punjabi O.S., Lalwani G.A. (2008). Macular Thickness Measurements in Normal Eyes Using Spectral Domain Optical Coherence Tomography. Ophthalmic. Surg. Lasers Imaging Retin..

[125] Asrani S., Edghill B., Gupta Y., Meerhoff G. (2010). Optical Coherence Tomography Errors in Glaucoma. J. Glaucoma.

[126] Cheung C.Y.L., Yiu C.K.F., Weinreb R.N., Lin D., Li H., Yung A.Y., Pang C.P., Lam D.S.C., Leung C.K.S. (2009). Effects of Scan Circle Displacement in Optical Coherence Tomography Retinal Nerve Fibre Layer Thickness Measurement: A RNFL Modelling Study. Eye.

[127] Wolf-Schnurrbusch U.E.K., Ceklic L., Brinkmann C.K., Iliev M.E., Frey M., Rothenbuehler S.P., Enzmann V., Wolf S. (2009). Macular Thickness Measurements in Healthy Eyes Using Six Different Optical Coherence Tomography Instruments. Investig. Ophthalmol. Vis. Sci..

[128] Krebs I., Hagen S., Brannath W., Haas P., Womastek I., de Salvo G., Ansari-Shahrezaei S., Binder S. (2010). Repeatability and Reproducibility of Retinal Thickness Measurements by Optical Coherence Tomography in Age-Related Macular Degeneration. Ophthalmology.

[129] Vizzeri G., Bowd C., Medeiros F.A., Weinreb R.N., Zangwill L.M. (2009). Effect of Signal Strength and Improper Alignment on the Variability of Stratus Optical Coherence Tomography Retinal Nerve Fiber Layer Thickness Measurements. Am. J. Ophthalmol..

[130] Wu Z., Vazeen M., Varma R., Chopra V., Walsh A.C., LaBree L.D., Sadda S.R. (2007). Factors Associated with Variability in Retinal Nerve Fiber Layer Thickness Measurements Obtained by Optical Coherence Tomography. Ophthalmology.

[131] Chen J.J., Thurtell M.J., Longmuir R.A., Garvin M.K., Wang J.-K., Wall M., Kardon R.H. (2015). Causes and Prognosis of Visual Acuity Loss at the Time of Initial Presentation in Idiopathic Intracranial Hypertension. Investig. Opthalmology Vis. Sci..

[132] Awadalla M.S., Fitzgerald J., Andrew N.H., Zhou T., Marshall H., Qassim A., Hassall M., Casson R.J., Graham S.L., Healey P.R. (2018). Prevalence and Type of Artefact with Spectral Domain Optical Coherence Tomography Macular Ganglion Cell Imaging in Glaucoma Surveillance. PLoS ONE.

